# Watching the days go by: Asymmetric regulation of caterpillar development by changes in photoperiod

**DOI:** 10.1002/ece3.7433

**Published:** 2021-03-18

**Authors:** Olle Lindestad, Inger M. Aalberg Haugen, Karl Gotthard

**Affiliations:** ^1^ Department of Zoology Stockholm University Stockholm Sweden

**Keywords:** alternative life‐history strategies, butterfly, developmental plasticity, diapause, phenotypic plasticity, photoperiodism

## Abstract

Many insects possess the plastic ability to either develop directly to adulthood, or enter diapause and postpone reproduction until the next year, depending on environmental cues (primarily photoperiod) that signal the amount of time remaining until the end of the growth season. These two alternative pathways often differ in co‐adapted life‐history traits, for example, with slower development and larger size in individuals headed for diapause. The developmental timing of these differences may be of adaptive importance: If traits diverge early, the potential for phenotypic differences between the pathways is greater, whereas if traits diverge late, the risk may be lower of expressing a maladaptive phenotype if the selective environment changes during development. Here, we explore the effects of changes in photoperiodic information during life on pupal diapause and associated life‐history traits in the butterfly *Pararge aegeria*. We find that both pupal diapause and larval development rate are asymmetrically regulated: While exposure to long days late in life (regardless of earlier experiences) was sufficient to produce nondiapause development and accelerate larval development accordingly, more prolonged exposure to short days was required to induce diapause and slow down prediapause larval development. While the two developmental pathways diverged early in development, development rates could be partially reversed by altered environmental cues. Meanwhile, pathway differences in body size were more inflexible, despite emerging late in development. These results show how several traits may be shaped by the same environmental cue (photoperiod), but along subtly different ontogenies, into an integrated phenotype.

## Graphical abstract

In many insects, winter diapause and nondiapause development comprise two plastically induced, alternative life‐history strategies. By subjecting butterfly larvae to controlled daylength regimes, we show that the co‐adapted traits comprising these strategies (diapause decision, body size, and growth rate) follow different ontogenies, and are differentially sensitive to changes in information during development.

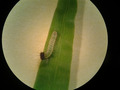

## 1 INTRODUCTION

Phenotypic plasticity allows organisms to thrive in environments that are variable in space and time (Stearns, 1989). A key source of environmental variability is the seasonal cycle, which brings changes in temperature, weather, food availability, and predation rates across the year. While often dramatic, seasonal changes can be predicted and anticipated through environmental signals, such as changes in the length of day versus night (Bradshaw & Holzapfel, 2007). Therefore, an organism may achieve high fitness at different times of year through seasonal plasticity: the controlled expression of seasonally appropriate body forms, physiologies, or behaviors (Moran, 1992; Shapiro, 1976; Varpe, 2017).

Plastic responses sometimes include suites of traits working together to form complex alternative strategies, as is seen with predator defense morphs in juvenile frogs (McCollum & Van Buskirk, 1996), paedomorphic versus metamorphic development in salamanders (Semlitsch et al., 1990), migratory polyphenism in locusts (Pener & Simpson, 2009), and indeed with environmentally controlled sex differentiation in many organisms (Ah‐King & Nylin, 2010). Such examples suggest that in the presence of an adaptive plastic response on one trait axis, selection can be expected to favor co‐adaptive fine‐tuning on additional trait axes.

A seasonal example of such multi‐trait phenotypes is provided by diapause/nondiapause polyphenism in insects. Many insects and other arthropods have the facultative ability to either enter diapause (a hormonally controlled resting state) at a given life stage, hence postponing reproduction until the following year, or develop directly to adulthood and attempt to reproduce (Tauber et al., 1986). The choice between these two pathways is controlled by seasonal cues such as photoperiod; diapause is the adaptive option when it is too late in the season to successfully reproduce, while nondiapause development, when possible, shortens the generation time, and thereby allows for multiplicative population increase. Across a range of insect species, the diapause/nondiapause binary trait is correlated with other core life‐history traits, such as body size and development rate. Although details vary between species (see Kivelä et al., 2013 for a comprehensive review), two common patterns are that individuals developing toward diapause also tend to show slower development rates (Friberg et al., 2012; Pöykkö & Hyvärinen, 2012; Välimäki et al., 2013) and/or mature at a larger final size (Aalberg Haugen & Gotthard, 2012; Fischer & Fiedler, 2001; Nylin, 1992).

Both of these patterns are consistent with adaptive predictions based on the amount of time stress placed on an individual relative to the end of the favorable season. From a given starting point in time, an individual destined for diapause can afford to develop slowly, while averting diapause and attempting to fit an additional reproductive cycle into the same year may necessitate faster development, implying rapid growth and/or a smaller adult size (Abrams et al., 1996). Modeling thus predicts that the optimal values for body size and growth rate (as well as other life‐history traits) are different for diapause‐destined and nondiapause‐destined individuals, and that these traits should diverge to form two alternative multi‐trait life‐history strategies (Kivelä et al., 2013). In addition, diapausing and nondiapausing individuals will experience different temporal environments as adults, which may also drive pathway differences in adult size (Van Dyck & Wiklund, 2002). Despite the apparent prevalence of these multi‐trait diapause/nondiapause phenotypes, little is known about their ontogeny, and how the different traits comprising them are regulated by environmental cues.

Of particular interest is the extent to which the set of traits constituting the diapause/nondiapause phenotypes are regulated independently of one another, how early in life trait regulation occurs, and to what extent trait regulation is reversible. One possibility is that, once induced by seasonal cues, individuals are irreversibly channeled into one of two discrete developmental programs, each with a corresponding set of trait values (Nijhout, 2003). As pointed out by Friberg et al., (2011), this would suggest an adaptive trade‐off: The earlier a pathway decision (i.e., to diapause or not) is made, the larger the scope for divergently expressing trait values (e.g., slow or fast development) that adaptively match either pathway; but the later a pathway decision is made, the higher the likelihood that the cues used to “decide” on a pathway accurately reflect the future selective environment.

Alternatively, diapause and its associated traits may be induced independently of one another, but by the same environmental cues (Mather, 1955), or pathway choice may be reversible by cues experienced later in development. While these latter scenarios would allow for more time to develop divergent trait values, they may also increase the risk of producing intermediate phenotypes of low fitness, through developmental instability or conflicting cues (DeWitt et al., 1998; Moran, 1992). Finally, there is the possibility that the expression of one trait indirectly affects the expression of another. In particular, plastic regulation of development rate early in the insect's life may determine which regulatory stimuli it becomes exposed to later on, hence phenotypes are molded into two overall responses under natural conditions (slow‐growing diapausers versus fast‐growing nondiapausers). This scenario would correspond to the “cascade”‐style developmental switch described by West‐Eberhard (2003).

The present study builds on previous findings in three butterfly species (*Pieris napi*, *Pararge aegeria*, and *Araschnia levana*), each from a lineage that has seemingly separately evolved diapause in the pupal stage. Friberg et al., (2011) showed that in all three species, the photoperiodic switch controlling whether or not to enter diapause is “locked in” relatively late in larval development. In other words, the daylength experienced last determined diapause decision, to a large extent overriding earlier experiences. However, this regulation was asymmetrically flexible: A decision to enter diapause could be reversed later in life than could a decision not to enter diapause, likely reflecting the relative amounts of time required to adequately prepare for each respective pathway. This result raises the following questions:

1. Do life‐history traits that tend to be co‐expressed with diapause, such as larval development rate and body size, also follow the same asymmetrically flexible pattern of induction?
2. When do differences in body size and development rate between the diapause and nondiapause pathways emerge—before or after the diapause decision is finalized?
3. Do changes in photoperiodic information during development alter the ontogenies of these co‐expressed life‐history traits?

Here, we address these questions in one of the three species studied by Friberg et al., *P. aegeria*, by systematically manipulating photoperiod regimes at different points during the larval period and observing the effect on the ontogeny of the diapause/nondiapause polyphenism.

## 2 MATERIALS AND METHODS

### 2.1 Study species

**FIGURE 1:**
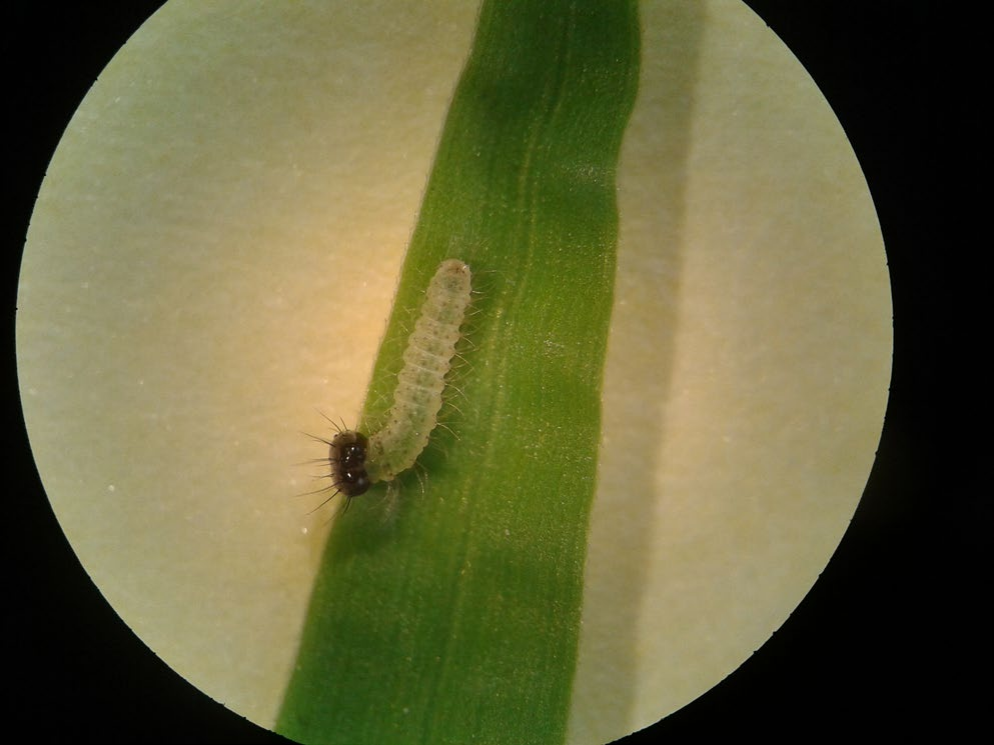
Newly hatched *Pararge aegeria* larva, within a few days of hatching, on a leaf of its host plant, bluegrass (*Poa annua*). Its black head capsule distinguishes it from later instars of the species, which are wholly green

*Pararge aegeria*, the speckled wood (Figure 1), is a woodland‐associated satyrine butterfly found across Eurasia. Its life cycle is complex, strongly shaped by photoperiod, and varies geographically with local climate (Nylin et al., 1989, 1995). In Sweden, bivoltine populations (i.e., producing two generations per year) exist in the south and on some Baltic islands (Lindestad et al., 2019), while northern mainland populations are univoltine (one generation per year). Generally speaking, pupal diapause is induced by short days during the larval period, while larvae exposed to long days enter nondiapause development. By definition, more or less all individuals born in a univoltine population go through diapause, but northern *P. aegeria* can still be made to go through nondiapause development in the laboratory, showing that they are kept from doing so in the wild by local adaptation of photoperiod thresholds (Lindestad et al., 2019). *P. aegeria* larvae headed for pupal diapause develop much slower, taking up to twice as long to pupate, compared to individuals in nondiapause development (Nylin et al., 1989). Body size differences also exist, with diapausing pupae being up to 10%–15% larger; however, this effect is limited to only some populations, seemingly depending on local voltinism patterns (Aalberg Haugen et al., 2012; Aalberg Haugen & Gotthard, 2015; Van Dyck & Wiklund, 2002). In addition to the nondiapause and pupal diapause pathways, *P. aegeria* is also capable of diapausing in the third larval instar, if exposed to very short days early in the larval period (Nylin et al., 1989). However, for the sake of simplicity, and because pupal diapause appears to be the dominant form of diapause both within (Wiklund & Friberg, 2011) and between Scandinavian populations (Gotthard & Berger, 2010; Wiklund et al., 1983), larval diapause will not be considered here.

### 2.2 Photoperiod experiment

*Pararge aegeria* for the experiment were derived from field‐mated females collected from three populations across Sweden in 2011. In late May/early June, females were collected from Stockholm (59.63°N, 18.52°E; univoltine population; 11 females) and Öland (56.62°N, 16.56°E; bivoltine population; 5 females); in August, females were collected from Skåne (56.29°N, 12.48°E; bivoltine population; 6 females). The experiment was carried out in two temporal blocks. The first block started in June, using first‐generation offspring of the wild females from Stockholm (6 families) and Öland (5 families). The second block started in September and used first‐generation offspring of the wild females from Skåne (6 families), alongside second‐generation offspring for Stockholm and Öland (1 family each). Apart from these differences, both experimental blocks used the same methods and were analyzed together. It should be noted that the difference in sampling time between populations (early versus late summer) complicates interpretation of population differences, as the genetic composition of the adult butterfly population may possibly change across the season.

**FIGURE 2:**
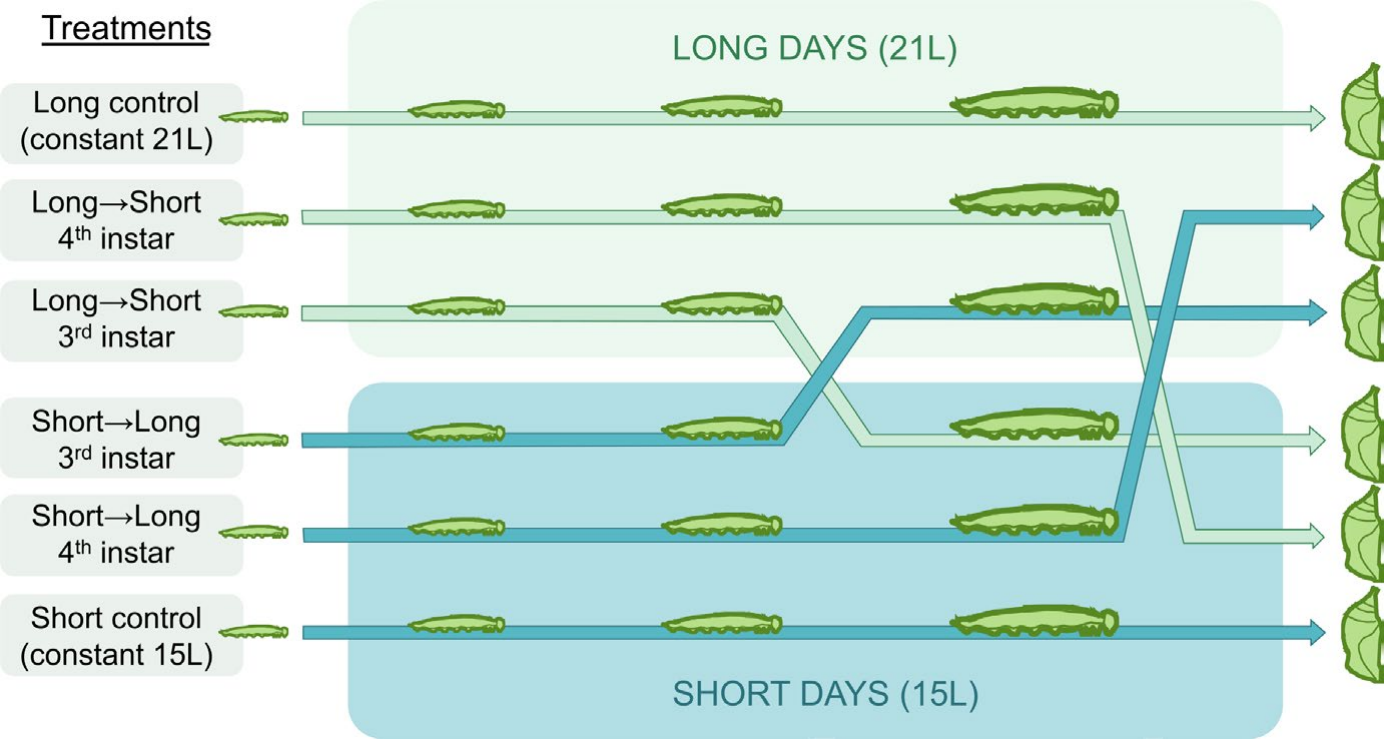
Schematic of experimental design, showing *Pararge aegeria* development through four larval instars to pupation. Larvae were divided between six treatments, consisting of exposure to short‐day or long‐day photoperiodic regimes at different points during larval development. Larvae were either exposed to constant daylengths (long control; short control), switched between daylength regimes (long to short; short to long) on the first day of the fourth larval instar, or switched between daylength regimes on the first day of the third larval instar

Shortly upon hatching from the egg, each larva was placed into an 0.5‐liter plastic container containing a living tuft of bluegrass (*Poa annua*). The grass in each rearing cup was replaced as needed, to ensure ad‐lib access to food throughout the experiment. Cups were placed into climate cabinets (Termaks series KB8400L; Termaks), set to 17°C, representing typical average diel temperature during summer in the studied region. The cabinet lights were programmed to one of two photoperiods: short days (15 hr light/9 hr dark) or long days (21 hr light/3 hr dark). Based on previous results (e.g., Lindestad et al., 2019), constant exposure to one of these two photoperiods reliably induces pupal diapause and nondiapause development (respectively) in the studied populations. Each photoperiod was duplicated, for a total of four cabinets. To test the effects of changes in daylength information during development, larvae were assigned into six treatments (Figure 2). The first two sets of larvae acted as control treatments: These were kept under constant daylength (either long or short) for the entire experiment. Another two sets of larvae were, upon molting into the third instar, moved to a cabinet set to the opposite daylength regime (from short to long, or long to short, respectively). The last two sets of larvae were likewise switched between daylength regimes, but not until later in development, at the molt to the fourth and final instar. After accounting for mortality, each combination of population and treatment was represented by 14–23 individuals (mean = 18.9). Eggs and larvae were checked daily, meaning the timing of hatchings and molts was precise to within a day. For *P. aegeria* larvae growing at a steady rate, all instars are roughly equivalent in length, meaning that the start of the third instar roughly corresponds to the mid‐point of the larval period (Nylin et al., 1989).

Experimental individuals were weighed at set points in development: on the day of hatching (to within 0.001 mg) using a Cahn 28 Electrobalance (Cahn Scientific), as well as on the day of molting to the third instar, on the day of molting to the fourth (final) instar, and two days after molting to the pupal stage (to within 0.1 mg), using a Precisa 205A balance (Precisa Gravimetric). The 2‐day wait for the pupae was to allow the pupal cuticle to harden, preventing damage during handling. For logistical reasons, the exact time (and hence also weight) at larval hatching could not be obtained for the Skåne population; however, all remaining data were recorded for this population as for the other two. Individuals were sexed according to the number of genital slits in the pupal cuticle, and pupal development was monitored to determine whether diapause had been initiated. At 17°C, a nondiapausing *P. aegeria* pupa is expected to develop within 25 days or less (Lindestad et al., 2020; Nylin et al., 1989); here, eclosion occurred either after <23 days or >45 days, allowing the two developmental pathways to be clearly separated.

### 2.3 Statistical analyses

The three primary response variables recorded from the experiment were diapause induction rate, development rate, and body weight. Variation in diapause induction was tested using a generalized linear model with a logit link function and diapause/nondiapause as the binary response variable. Population, sex, and treatment (six‐level factor; see Figure 2) were used as explanatory variables.

Development rate was defined as 1/*d*, where *d* is the time needed to complete a given stage of development. Three intervals were separately analyzed: the time from hatching to the second molt (instars 1 + 2; these data only available for Öland and Stockholm), the time from the second molt to the third molt (instar 3), and the time from the third molt to pupation (instar 4). For each of these analyses a three‐way ANOVA was used, with treatment, sex, and population as explanatory variables. In one of the six treatments (larvae that had been switched from long to short days in the third instar), development rate in the fourth instar in particular was strongly bimodal according to diapause decision (Figure S1). For this reason, this treatment was split by diapause decision, giving seven treatment levels instead of six, when analyzing fourth‐instar development rate.

Finally, weight was analyzed as a repeated measurement, using a mixed linear model with individual treated as a random effect. Developmental stage (third instar/fourth instar/pupa), treatment (six levels), sex, and population were used as fixed effects, hence testing for differences in weights between treatments at different points in development. Because larvae grow by several orders of magnitude, weights were log‐transformed in order to scale values across the time axis.

All analyses were carried out in R version 3.6.1 (R Development Core Team, 2019). For each analysis, all fittable two‐ and three‐way interactions between the explanatory variables were tested, and nonsignificant interactions were removed stepwise (in order of highest p‐value) so as not to sacrifice statistical power (Engqvist, 2005). The significance of model terms (*α* = .05) was evaluated using analysis of variance (for continuous responses, i.e., weight and development rate) or analysis of deviance (for binomial responses, i.e., diapause) with the ANOVA function from the *car* package (Fox & Weisberg, 2019). The final models are shown in Table S1 and Table S2. Because larvae were shifted between photoperiod regimes as they developed, the actual number of unique conditions experienced was two, then four, then six, depending on the stage of the experiment (Figure 2). To address this, planned contrasts were applied to the final models for development rate and weight, in order to pool and compare larvae that had experienced the same conditions up until a given point. At the start of the third instar, the only contrast was long days versus short days. At the start of the fourth instar, long versus short days were contrasted, and larvae that had switched photoperiods in the previous instar were additionally contrasted with their respective photoperiod of origin. At pupation, all six treatments were distinct, so all pairwise comparisons were made, using Tukey's HSD method to compensate for multiple testing. All contrasts were applied using the *emmeans* package (Lenth, 2020) and were calculated without controlling for population except where stated otherwise. All treatment contrasts are summarized in Table S3–Table S6.

## 3 RESULTS

### 3.1 Diapause induction

**FIGURE 3:**
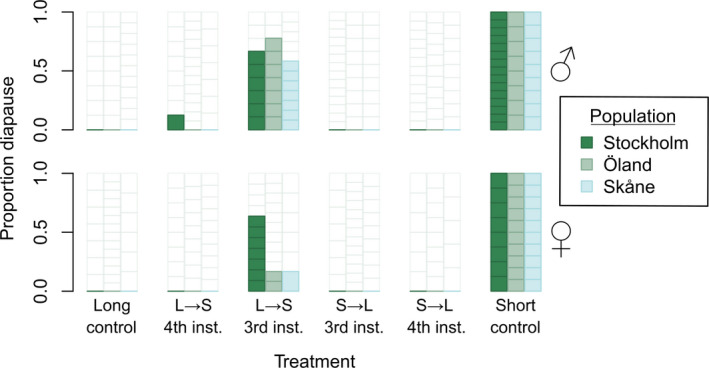
Diapause induction rates per sex (above, males; below, females), population and treatment (S = short days; L = long days; “→” = change in daylength regime, at the start of either instar 3 or instar 4). Bar segments show sample sizes (closed segments for diapause individuals; open segments for nondiapause individuals)

The photoperiods experienced during the larval period strongly affected the induction of pupal diapause (analysis of deviance; treatment χ^2^_5_ = 312; *p* < .001), with asymmetric results of switching daylength regimes. Constant exposure to short days (15 hr) resulted in 100% diapause induction, whereas 0% diapause induction was attained in all three treatments that ended with larvae experiencing long days (21 hr) (Figure 3). In other words, exposure to long days in the fourth and final instar was sufficient for consistently activating nondiapause development, regardless of previously experienced daylength. Meanwhile, the opposite change in daylength, from long to short days, only resulted in approximately half of individuals entering pupal diapause, even when switched as early as the third instar. Diapause patterns were similar across populations (analysis of deviance; population χ^2^_2_ = 5.31; *p* = .07); there were no significant interactions with other explanatory factors. Males were more likely to enter diapause overall (analysis of deviance; sex χ^2^_1_ = 8.83; *p* = .003), as is common in butterflies (Wiklund et al., 1992). A single male from the univoltine Stockholm population was the only individual that entered diapause upon being switched from long to short days in the fourth instar.

### 3.2 Development rate

**FIGURE 4:**
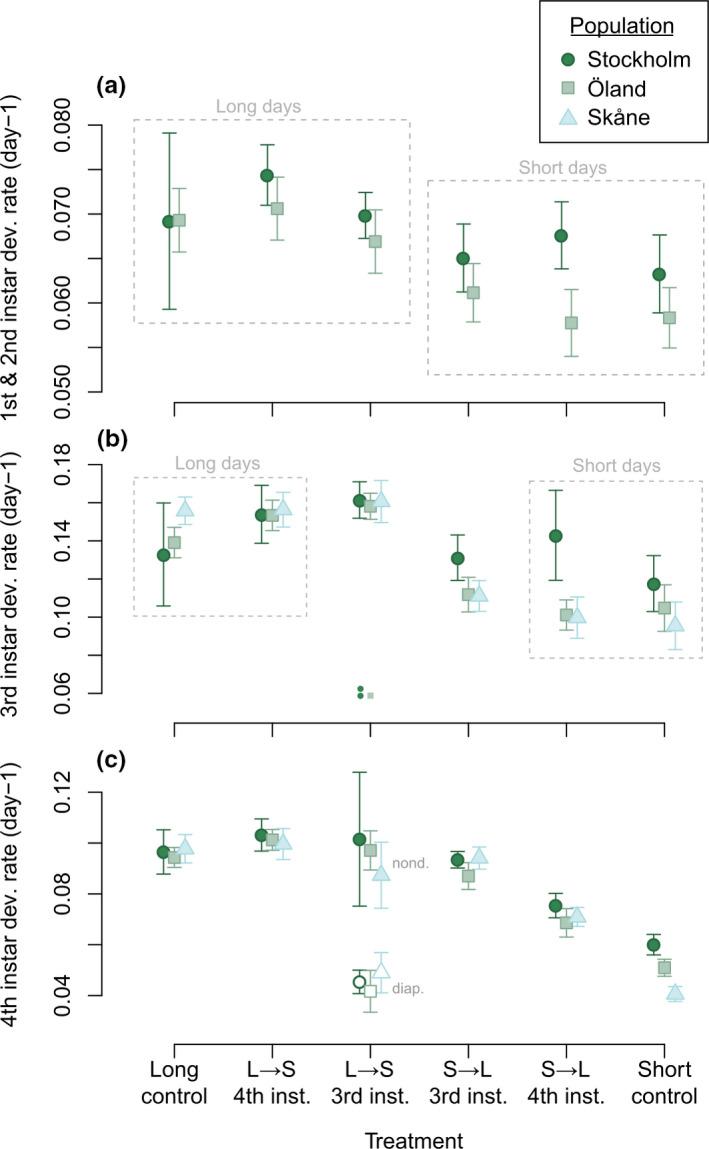
Mean development rate (± 95% CI) by treatment and population. (a) First and second instar; (b) third instar; and (c) fourth instar. Treatments joined by dashed rectangles are those that had not yet diverged in the experimental protocol, and hence had experienced the same conditions up until the point that the data was recorded. Males and females are pooled for all panels, as the overall pattern was similar for both sexes. Treatment abbreviations: S → L, short days to long days; L → S, long days to short days

Larval development rates were dependent on photoperiod regime, with larvae of all populations developing faster under constant long days than under constant short days (Figure 4). Although subtle at first, the effect of daylength was detectable early in life: The molt to the third instar occurred on average two days later under short days than under long days (planned contrast: t_186_ = 7.79, *p* < .001). This difference was magnified later during development, with the fourth instar typically taking nearly twice as long to complete for short‐day control larvae than for long‐day control larvae (planned contrast: t_281_ = 23.6; *p* < .001). Development rate results were complex, largely owing to sex differences: In *P. aegeria*, larvae not headed for diapause are sexually dimorphic for development rate (Nylin et al., 1993), but this effect is only found in bivoltine populations (Aalberg Haugen & Gotthard, 2015). Hence, a three‐way interaction was seen in the fourth instar (analysis of variance; sex × treatment × population F_12_ = 1.95; *p* = .03). However, this contributed comparatively little to the overall models (Table S1); by far the largest amount of variation in development rate, especially in the fourth and final instar, was explained by the overall effect of photoperiod treatment, which we will now describe in detail.

Much like with diapause induction, when larvae experienced a change in daylength during development, the effects on development rate were asymmetric depending on the direction of change, and also depended greatly on the timing of the change. In the third instar (Figure 4b), larvae that had recently been moved from short days to long days showed slightly increased development rates relative to larvae that remained in short days (planned contrast; t_261_ = 3.31, *p* = .0011). A decrease in daylength, on the other hand, had little immediate effect on average; if anything, development was slightly faster than in the remaining long‐day larvae (planned contrast; t_261_ = 2.60; *p* = .0099). However, three individuals in this group instead showed drastically lowered development rates (two from Stockholm, one from Öland; all three later entered diapause). These three extreme outliers (more than 3 IQR below the first quartile) were excluded from the linear model for third‐instar development rate, as they likely represent a biologically distinct response, but are shown as separate points in Figure 4b (complete raw data for this trait are shown in Figure S1).

A similar but stronger short‐term pattern was seen when the photoperiod change instead occurred in the fourth instar. Again, lengthening days in the fourth instar sped up development (Figure 4c), leading to fourth‐instar development rates intermediate between those for the long‐ and short‐day controls groups (Tukey contrast; t_281_ = 10.81; *p* < .001). A decrease in daylength did not result in a lower development rate, unlike what may be expected; on the contrary, a slight increase was seen relative to the long‐day control group (Tukey contrast; t_281_ = 3.23; *p* = .023). Finally, the most dramatic effects on fourth‐instar development rate were observed in those larvae that had experienced a photoperiod switch in the third instar. Larvae that had experienced an increase in daylength had now fully adjusted their phenotype, and developed at a rate indistinguishable from that of the long‐day control larvae. Meanwhile, larvae that had experienced a decrease in daylength in the third instar showed a strongly bimodal response, which correlated closely with whether diapause occurred after pupation: those not headed for diapause developed fast, while those headed for diapause reversed their previous response and instead developed very slowly, mirroring their short‐day control‐group counterparts. In contrast, only a weak correlation between development rate and the eventual diapause decision could be observed for this treatment in the third instar (Figure S1).

**FIGURE 5:**
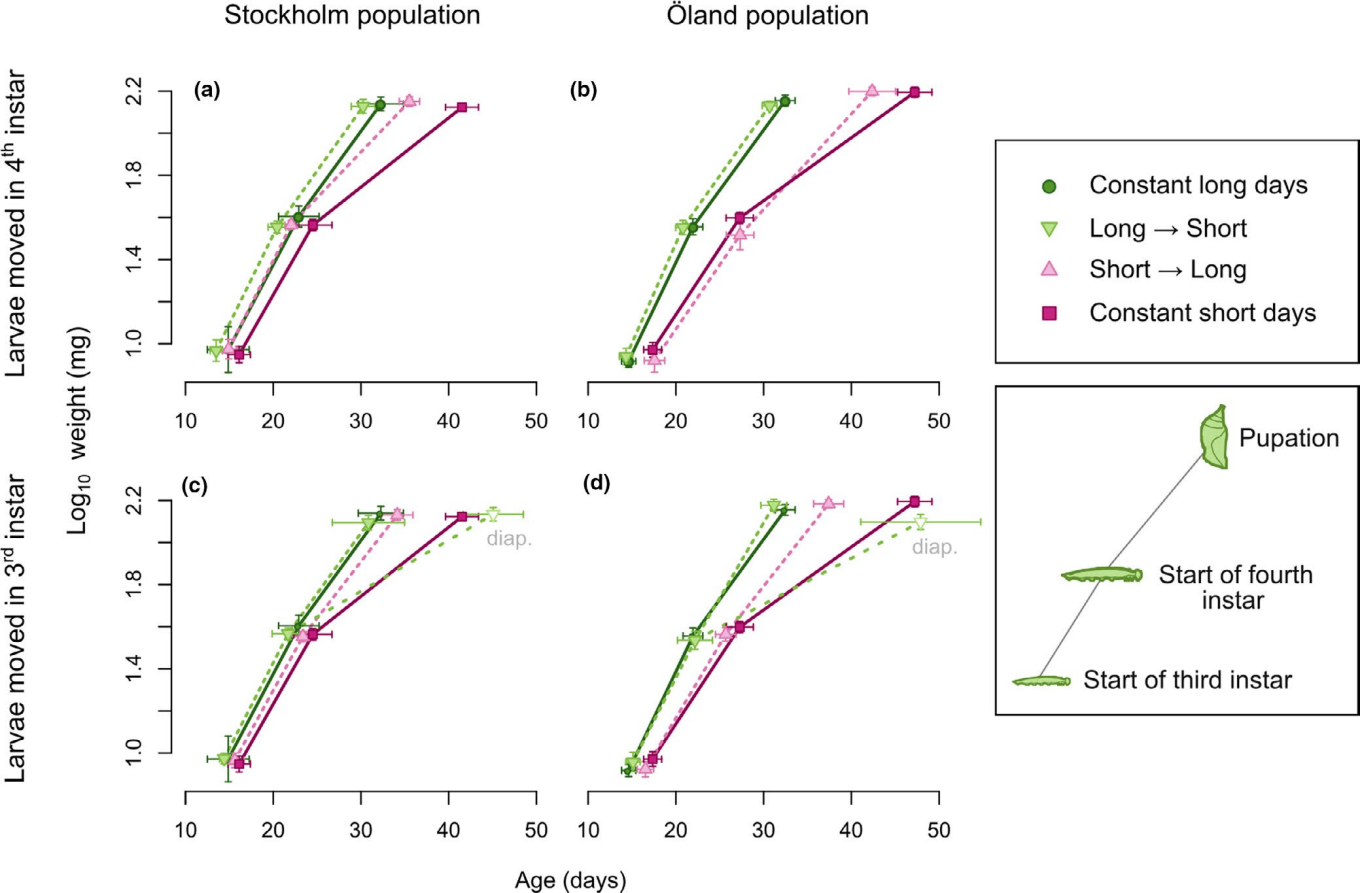
Growth trajectories from the start of the third instar to pupation, showing mean age and weight at each molt (sexes pooled). Bars show 95% confidence intervals on both axes. Top row: results for larvae switched between daylength regimes in the fourth instar; bottom row: results for larvae switched in the third instar (the same per‐population values for the control/constant daylength treatments are displayed for reference in both rows). In c and d, the fourth‐instar trajectory for the long‐to‐short treatment is split by diapause decision, with individuals that entered diapause shown as open downward triangles and marked “diap.” The Skåne population is not shown, as the lack of precise hatching dates meant that age could not be calculated

The overall outcomes of the regulation of development rate across the whole larval period are shown in Figure 5. Short‐day control larvae pupated considerably later than long‐day control larvae, with an average difference of 46% for Öland and 29% for Stockholm, respectively (no data for Skåne, as hatching dates were not recorded). Larvae switched from short to long days in the fourth instar ended up with intermediate pupation dates, that is, partially compensating for slow early development, while larvae switched from long to short days in the fourth instar pupated at a very similar age to their long‐day control counterparts (Figure 5a‐b). When the move from short to long days occurred as early as the third instar, larvae were much better able to adjust their phenotype (Figure 5c‐d). This was especially true for Stockholm, as this population showed a relatively small baseline difference between long‐day and short‐day development rates. Finally, larvae switched from long to short days in the third instar had very different outcomes depending on diapause decision: Individuals headed for diapause pupated at times similar to the short‐day control group, while those not headed for diapause pupated at times similar to the long‐day control group.

### 3.3 Weight accumulation

Compared to the results for development rate, the effects of photoperiod treatment on weight were weaker, and quite population‐dependent (analysis of deviance; population × treatment χ^2^_10_ = 22.5; *p* = .012). The only population that showed a significant difference in final size between the control treatments was Öland, where constant exposure to short days produced pupae that were approximately 10% heavier than did constant exposure to long days (within‐population planned contrast; t_567_ = 4.01, *p* = .001). This difference was not detectable at any earlier life stage. In contrast to the results for development time, changes in photoperiod regime during development did not appear to drive final size; instead, pupal weights tended to correspond to the initial photoperiod experienced (Figure 5; Figure S2). Females were larger than males across all populations (analysis of deviance; sex χ^2^_1_ = 142.1; *p* < .001); unlike the late effects of photoperiod, the sex difference was detectable as early as the third instar and was increased further in later stages.

## 4 DISCUSSION

Consistent with earlier findings from both *P. aegeria* and other species (Friberg et al., 2011), photoperiodic control of the diapause/nondiapause developmental switch is seen to be asymmetrically flexible: Activating diapause development required a consistent photoperiod signal for a longer time than activating nondiapause development (Figure 3). Building on these findings, we here additionally show that a similar asymmetry exists in prediapause development rate, a trait that has likely been under selection to match the diapause phenotype (Kivelä et al., 2013). An increase in daylength led to an immediate increase in development rate (further accelerated by prolonged exposure to long days), favorable for producing an additional generation; in contrast, the slow development typically seen in diapause‐destined larvae was here only engendered by sustained exposure to short days (Figure 4, Figure 5). The results are consistent with *P. aegeria* possessing two alternative, overall modes of larval development, cued by photoperiod: slow, “diapause‐track” development and fast, “non‐diapause‐track” development. It is evident that, while these two modes diverge early in life, they are not irreversibly locked states; instead, photoperiodic information is continually used throughout the larval period to update developmental plasticity. Switching between developmental modes does not appear to be instantaneous, but shows a degree of inertia, as the phenotype matching the new photoperiodic environment did not, in most individuals, fully manifest until the next larval instar after the change in photoperiod had taken place (Figure 4). This inertia may represent a delay in perceiving and acting on the changed photoperiod signal, and/or a delay in “resetting” the hormonal machinery that controls development and growth.

Meanwhile, a rather different result was obtained for another life‐history trait associated with the diapause switch: body size. Here, differences between individuals reared under short versus long days were not visible until the pupal stage, suggesting that these interpathway differences, unlike those seen for development rate, do not emerge until late in larval development.

### 4.1 Asymmetric regulation of diapause and development rate

The observed pattern, where a decision to enter diapause can be reversed by a changed photoperiod signal later in life than can a decision *not* to enter diapause, may reflect a constraint in the ability of an insect to enter diapause unprepared (Friberg et al., 2011). Diapause tends to be a long‐lasting and demanding state during which an insect is subject to adverse conditions such as extreme cold and drought, necessitating protective adaptations (Danks, 2000; Denlinger, 1991). Furthermore, a diapausing insect (especially a pupa) often has little or no access to food, and must therefore rely on resources gathered before diapause (Hahn & Denlinger, 2007, 2011). Although metabolism is low during diapause, this does not mean that no resources are consumed: *P. aegeria* pupae may lose up to 5% of their mass during winter (Lindestad et al., 2020). Hence, if the physiological preparations required for successful diapause take a longer time to establish than those required for nondiapause development, it may be adaptive to resist a sudden switch to diapause development, even given environmental cues signaling the end of the season. However, the metabolomic similarity of diapause‐destined and nondiapause‐destined larvae seen in some species (Kivelä et al., 2019) speaks against this hypothesis. The asymmetric pattern may also be a result of other selective drivers, such as the multiplicative increase afforded by an additional generation, or the risks associated with entering diapause too early.

Many insect species quantitatively regulate development rate in response to photoperiod (reviewed by Beck, 1980; examples in Shindo & Masaki, 1995; Gotthard, 1998; Gotthard et al., 1999; Strobbe & Stoks, 2004; Shama & Robinson, 2006). Development rate variation shows a clear connection to time constraint: Photoperiods that signal seasonal progression (shorter days in the summer and fall; longer days in the spring) tend to speed up development, preventing the insect's life cycle from drifting out of sync with the changing environment or with conspecifics (Gotthard et al., 2000; Shindo & Masaki, 1995). In insects with the potential for more than one generation per year, these time horizons are more complicated, as a decision not to diapause imposes the additional time stress of fitting an additional generation into the remainder of the season (Kivelä et al., 2013; Roff, 1980). Accordingly, in *P. aegeria*, the long daylengths associated with nondiapause development are also associated with highly accelerated development and growth, and just as a change from short to long days was able to avert diapause, it also caused development to accelerate despite slow development earlier in life (Figure 4).

While lengthening days always had the effect of speeding up development, the effect of shortening days was more complex. Shortening days in the fourth instar actually produced a slight *increase* in development rate (Figure 4c), resulting in two days earlier pupation on average (Figure 5). It is difficult to say whether this small boost is adaptive, or merely a physiological quirk. Responding to a drop in daylength at the end of the larval period by speeding up development may well improve fitness: Larvae in this treatment are too far gone to switch to diapause development (Figure 3), so if shortening days signal the approaching end of the season, adulthood should be attained fast instead, hence “making the best of a bad situation”. A similar boost was visible when days shortened in the third instar, suggesting that it is a general short‐term effect (Figure 4b), although the effect was later strongly reversed in those individuals that, as the short days continued, switched to diapause‐track development and slowed down their development accordingly.

It should be noted that the photoperiods used here (21 versus 15 hr light) are extremes that serve as unambiguous diapause/nondiapause signals for all three studied populations. Laboratory exposure of *P. aegeria* larvae to constant, intermediate daylengths often produces individuals with a greatly extended larval period (up to three months) that nonetheless do not enter diapause at pupation, indicating that the development rate polyphenism and the diapause polyphenism are in fact at least semi‐distinct on a physiological level, and have subtly different photoperiod thresholds (Lindestad et al., 2019; Nylin et al., 1989). But even if the two plastic switches operate semi‐independently, the gradual change in daylength that will naturally occur across the season should differentially canalize the responses into distinct phenotypes. Larvae hatched late in the season will experience short daylengths and develop slowly, hence exposing them to even shorter daylengths later in life, and successfully inducing pupal diapause. Larvae hatched around the summer solstice will undergo sustained exposure to long days, leading to fast development to adulthood without diapause. For larvae hatched significantly before the solstice (which will be more common at lower latitudes), the effect will presumably be a synchronization of the nondiapausing cohort: Early‐hatched larvae may develop slowly at first, but gradually lengthening days will speed up development (and avert pupal diapause) to match larvae born later. These interactions between photoperiodic control of diapause and photoperiodic control of prediapause development rate, which have been reproduced in simulations (Lindestad et al., 2019), exemplify how developmental plasticity at different stages in an organism's life can self‐reinforce or modulate other plastic traits in a cascade fashion (West‐Eberhart, 2003).

### 4.2 Body size and diapause decision

Accumulating the materials to build an adult body takes time; an organism reaching adulthood within a shorter period of time must therefore either mature at a smaller size, compensate for the lost growing time by accumulating mass at a faster rate, or some combination thereof (Abrams et al., 1996; Davidowitz & Nijhout, 2004). *P. aegeria* skews strongly toward the latter option: the considerable variation in development rates between the treatments was matched to a large extent by variation in growth rates (defined as average weight gain per unit time) (Figure S1), resulting in comparatively small variation in final size.

To the extent that body size differences between treatments emerged, they were mostly found in the Öland population, as expected based on earlier common‐garden results for these populations (Aalberg Haugen et al., 2012; Aalberg Haugen & Gotthard, 2015). The difference in body size between long‐day and short‐day Öland individuals was not detectable at any life stage earlier than pupae (Figure S2), suggesting that it emerges at some point during the fourth instar. (An apparent difference between long‐day and short‐day individuals can be seen as early as the third instar in Figure 5, but this was due to a coincidental hatching size difference between the two control groups; no overall photoperiod effect was seen when considering all six treatments groups.) These results are reminiscent of those obtained in scarce swallowtail butterflies, where the size polyphenism is reversed (nondiapause individuals are larger), but the size difference arises from higher growth rates only at the end of the last larval instar (Esperk et al., 2013). Intensive studies of the moth *Manduca sexta* have revealed that body size is determined by the interplay of three parameters: basal growth rate, a critical weight, and the delay period from when a larva reaches the critical weight to when the resulting hormonal cascade arrests growth and triggers preparations for the molt to the pupal stage (Callier & Nijhout, 2013; Davidowitz & Nijhout, 2004; Nijhout et al., 2006). While each larval instar increases in size by the same multiple, so that molts between instars occur at predictable weights, the final instar “overshoots” the critical weight by continuing to grow during the delay period (Nijhout et al., 2006). If *P. aegeria* functions along similar lines, it is possible that the plastic size difference between developmental pathways is achieved by modulating the length of the delay period; this would leave the size at each previous molt the same for both pathways, as was observed.

When comparing the results for body size to those obtained for larval development rate, an apparent paradox emerges. One may expect that, if the size difference is only established late in the larval period, it should be responsive to adjustment by changes in photoperiod earlier during life. However, this was not observed: Final sizes tended to correspond to the initial photoperiod regime, and Öland individuals switching to diapause development in response to shortening days pupated at a smaller size than those reared in constant short days (Figure 5). In other words, body size diverged between photoperiods later in life, but was nonetheless *less* adjustable to the environmental conditions experienced late in life, than development rate.

A possible interpretation is that the between‐pathway size divergence that occurs in the fourth instar is driven by physiological mechanisms that are primed earlier during life, resulting in a delayed and inflexible effect of photoperiod on weight accumulation. Growth rates in general were seen to be highly flexible and responsive to photoperiod treatment (Figure S1b), but if the diapause‐pathway size difference utilizes a separate mechanism (as discussed above), this need not be a contradiction, and may explain how the development rate polyphenism can be shared across Scandinavian populations (Lindestad et al., 2019) while the body size polyphenism is not (Aalberg Haugen & Gotthard, 2015). Another possible explanation is that subjecting larvae to such unnaturally drastic shifts in daylength (and hence imposing rapid shifts in developmental strategy) resulted in physiological stress, which may have manifested as decreased final size, at least in individuals forced to change to the more resource‐demanding diapause pathway. As growth rate was only coarsely measured here, with a single weighing per instar, a more detailed investigation of these mechanisms will need to await further study.

## 5 CONCLUSIONS

Here, we have compared the ontogeny of three plastically correlated insect traits: diapause/nondiapause, development rate, and final body size. Results show that development rate in *P. aegeria* responds to photoperiod early in life, long before the diapause decision is finalized, which allows for large differences in the final phenotype (i.e., age at maturity). However, development rate also continually responds to changes in photoperiodic information, following the same asymmetric pattern of sensitivity seen for the diapause/nondiapause trait. As photoperiod changes across the season, current development rate affects future exposure to photoperiodic signals; hence, development rate forms part of a developmental cascade shaping the growth trajectory of an individual. In contrast to development rate, body size regulation appears to diverge late in life and did not show the same flexible response to changes in photoperiod. These results underscore how coordinated phenotypes like the diapause/nondiapause alternative pathways can evolve from suites of traits that share a cue (photoperiod), but have different ontogenies.

## CONFLICT OF INTEREST

None declared.

## AUTHOR CONTRIBUTIONS

**Olle Lindestad:** Data curation (lead); Formal analysis (lead); Software (lead); Visualization (lead); Writing‐original draft (lead); Writing‐review & editing (equal). **Inger Marie Aalberg Haugen:** Conceptualization (equal); Investigation (lead); Methodology (equal); Writing‐original draft (supporting). **Karl Gotthard:** Conceptualization (equal); Funding acquisition (lead); Investigation (supporting); Methodology (equal); Project administration (lead); Supervision (lead); Writing‐review & editing (supporting).

## Supporting information

Appendix S1

## Data Availability

The full dataset of the study (laboratory results and analysis scripts) has been made available through Dryad Digital Repository at https://doi.org/10.5061/dryad.bzkh1897w
