# Supplementary material for: Watching the days go by: Asymmetric regulation of caterpillar development by changes in photoperiod
Source: Ecol Evol. 2021 Mar 18;11(10):5402–12. doi: 10.1002/ece3.7433 (PMC8131801; doi:10.1002/ece3.7433)
Supplement: Supplementary file 1 — Appendix S1 [file ECE3-11-5402-s001.docx]

**Supplementary figures and tables**

for manuscript **“**Watching the days go by: asymmetric regulation of caterpillar development by changes in photoperiod”, submitted to *Ecology and Evolution*, February 2021

**
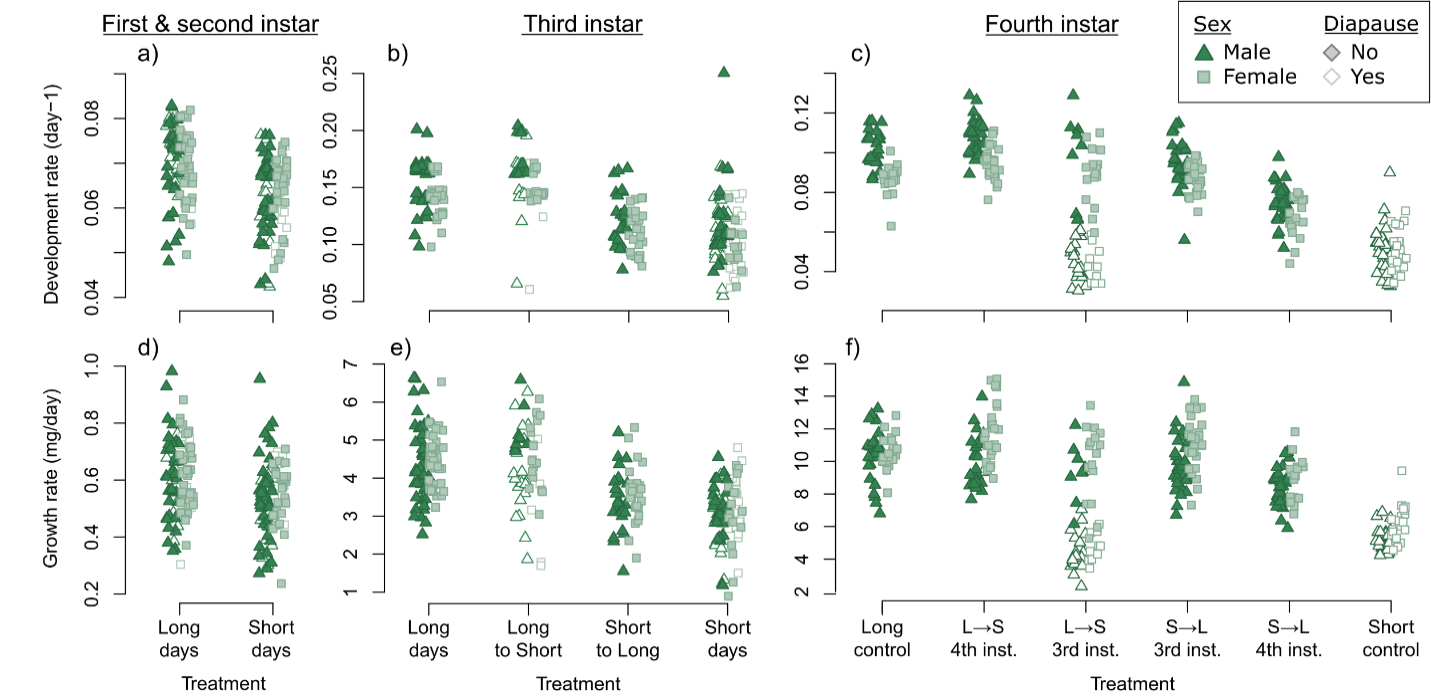
**

**Figure S1.** Raw data (points jittered somewhat for visibility) for development rate (top) and growth rate (bottom) across all three populations. Left column (a, d) shows results for instars 1 and 2; middle column (b, e) shows results for instar 3; right column (c, f) shows results for instar 4. Males are shown as triangles; females are shown as squares. Open symbols represent individuals that went on to enter diapause in the pupal stage. Note that in panels a, b, d and e, overlapping treatments have been pooled: “long days” and “short days” refer to both control larvae and larvae due to be switched between photoperiods in later instars, hence the mix of diapause responses.


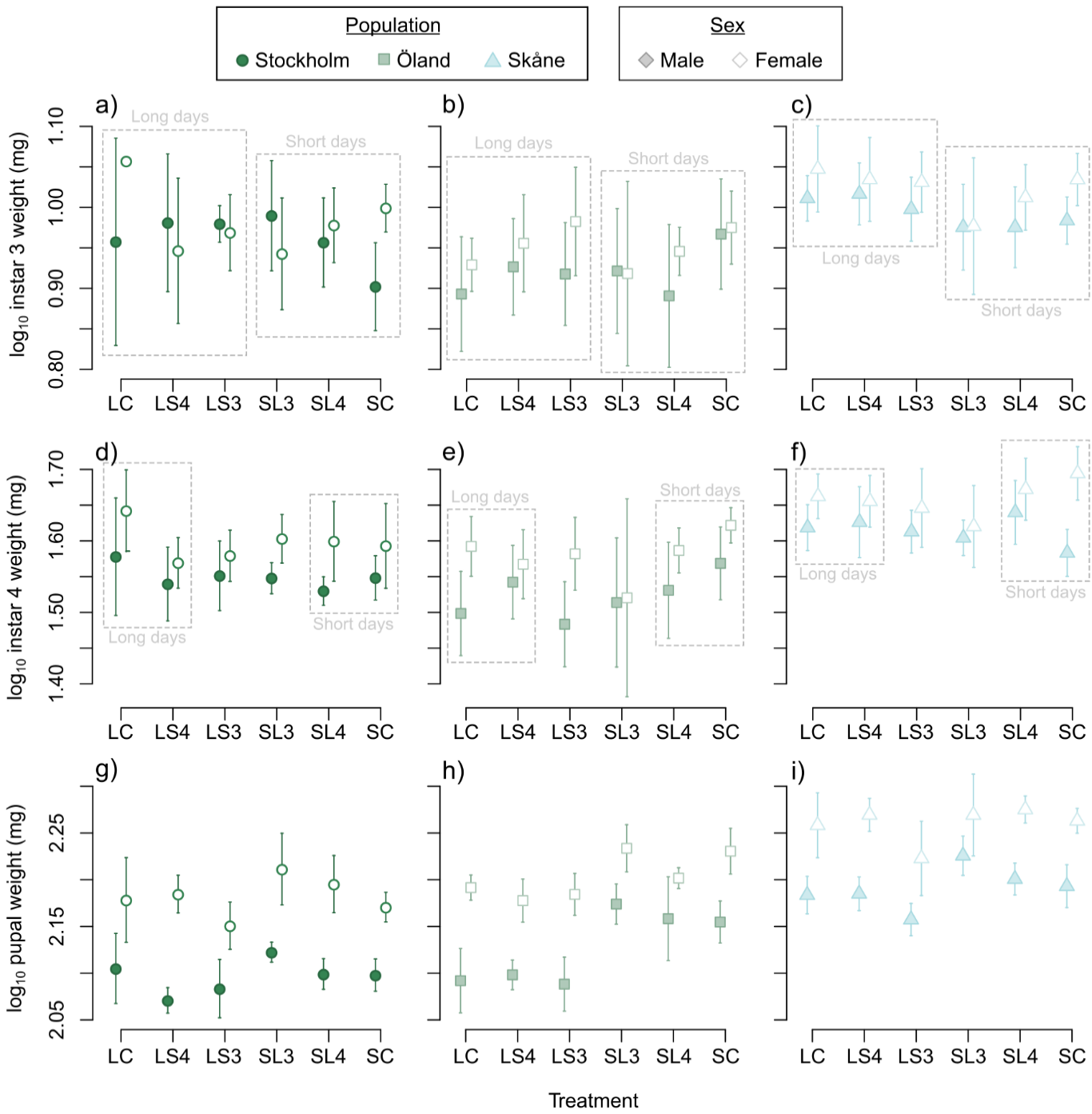


**Figure S2.** Mean weight (±95% CI) per sex and population at different points in development: at the start of the third instar (a-c); at the start of the fourth instar (d-f); two days after pupation (g-i). Closed symbols show males; open symbols show females. Dashed rectangles in a-f represent treatments that had not yet diverged in the experimental protocol, and hence had experienced the same conditions up until the point that the data was recorded. Treatment abbreviations reflect the order of photoperiod regimes and in which instar the photoperiod changed: LC, long control; SC, short control; LS3, long to short in 3^rd^ instar; LS4, long to short in 4^th^ instar; SL3, short to long in 3^rd^ instar; SL4, short to long in 4^th^ instar.

**Table S1.** Analysis of variance tables for the final linear models (i.e. after the removal of nonsignificant interactions) used to analyze development rate.

| **Response** | **Model term** | **Sum of squares** | **Mean squares** | **df** | **F** | **p** |
| --- | --- | --- | --- | --- | --- | --- |
| Instars 1+2 dev. rate | Treatment (six groups) | 0.0035 | 0.00070 | 5 | 13.19 | <0.001 |
|  | Sex | 0.0000012 | 0.0000012 | 1 | 0.022 | 0.88 |
|  | Population (Öland/Stockh.) | 0.00085 | 0.00085 | 1 | 15.93 | <0.001 |
|  | Residual | 0.0099 | 0.000053 | 186 | - | - |
| Instar 3 dev. rate | Treatment (six groups) | 0.13 | 0.026 | 5 | 65.46 | <0.001 |
|  | Sex | 0.012 | 0.012 | 1 | 29.88 | <0.001 |
|  | Population (all) | 0.0085 | 0.0042 | 2 | 10.60 | <0.001 |
|  | Treatm × pop | 0.019 | 0.0019 | 10 | 4.82 | <0.001 |
|  | Residual | 0.10 | 0.00040 | 261 | - | - |
| Instar 4 dev. rate | Treatment (seven groups) | 0.14 | 0.023 | 6 | 274.11 | <0.001 |
|  | Sex | 0.0058 | 0.0058 | 1 | 69.20 | <0.001 |
|  | Population (all) | 0.0018 | 0.00089 | 2 | 10.56 | <0.001 |
|  | Treatm × sex | 0.0029 | 0.00048 | 6 | 5.70 | <0.001 |
|  | Treatm × pop | 0.0042 | 0.00035 | 12 | 4.16 | <0.001 |
|  | Sex × pop | 0.00012 | 0.000062 | 2 | 0.73 | 0.48 |
|  | Treatm × sex × pop | 0.0020 | 0.00016 | 12 | 1.95 | 0.029 |
|  | Residual | 0.024 | 0.000084 | 281 | - | - |

**Table S2.** Analysis of deviance tables for the final linear models (i.e. after the removal of nonsignificant interactions) used to analyze diapause induction rate and weight, respectively.

| **Response** | **Model term** | **χ^2^** | **df** | **p** |
| --- | --- | --- | --- | --- |
| Diapause induction | Sex | 8.83 | 1 | 0.0030 |
|  | Treatment | 312.01 | 5 | <0.001 |
|  | Population | 5.31 | 2 | 0.070 |
| Weight (repeated measures) | Dev. stage | 86974.80 | 2 | <0.001 |
|  | Treatment | 7.21 | 5 | 0.21 |
|  | Population | 223.12 | 2 | <0.001 |
|  | Sex | 142.14 | 1 | <0.001 |
|  | Dev. stage × treatment | 52.90 | 10 | <0.001 |
|  | Dev. stage × population | 45.20 | 4 | <0.001 |
|  | Dev. stage × sex | 39.41 | 2 | <0.001 |
|  | Treatment × population | 22.54 | 10 | 0.013 |

**Table S3.** Treatment contrasts for development rate, averaged over sexes and populations. For the models analyzing instars 1+2 and instar 3, planned contrasts were used to handle shared photoperiod regimes between treatment groups. For the model analyzing instar 4, all pairwise treatment contrasts were analyzed using Tukey’s method. Treatment abbreviations reflect the order of photoperiod regimes and in which instar the photoperiod changed: LC, long control; SC, short control; LS3, long to short in 3^rd^ instar; LS4, long to short in 4^th^ instar; SL3, short to long in 3^rd^ instar; SL4, short to long in 4^th^ instar.

| **Response variable** | **Contrast** | **Difference** | **SE** | **df** | **T** | **p** |
| --- | --- | --- | --- | --- | --- | --- |
| Instar 1+2 dev. rate | Long days — short days | 0.0083 | 0.0011 | 186 | 7.79 | < 0.001 |
| Instar 3 dev. rate | Long days — short days | 0.038 | 0.0031 | 261 | 12.39 | < 0.001 |
|  | Long days — LS3 | -0.012 | 0.0037 | 261 | -3.31 | 0.0011 |
|  | Short days — SL3 | -0.009 | 0.0035 | 261 | -2.60 | 0.0099 |
| Instar 4 dev. rate | LC — LS3(diap) | 0.052 | 0.0026 | 281 | 19.90 | <.0001 |
|  | LC — LS3(nond) | -0.0031 | 0.0026 | 281 | -1.17 | 0.90 |
|  | LC — LS4 | -0.0061 | 0.0019 | 281 | -3.23 | 0.023 |
|  | LC — SC | 0.045 | 0.0019 | 281 | 23.63 | <.0001 |
|  | LC — SL3 | 0.0037 | 0.0019 | 281 | 1.99 | 0.42 |
|  | LC — SL4 | 0.025 | 0.0019 | 281 | 12.74 | <.0001 |
|  | LS3(diap) — LS3(nond) | -0.055 | 0.0031 | 281 | -17.56 | <.0001 |
|  | LS3(diap) — LS4 | -0.058 | 0.0026 | 281 | -22.76 | <.0001 |
|  | LS3(diap) — SC | -0.0074 | 0.0026 | 281 | -2.90 | 0.061 |
|  | LS3(diap) — SL3 | -0.048 | 0.0025 | 281 | -19.14 | <.0001 |
|  | LS3(diap) — SL4 | -0.027 | 0.0026 | 281 | -10.59 | <.0001 |
|  | LS3(nond) — LS4 | -0.0031 | 0.0026 | 281 | -1.18 | 0.90 |
|  | LS3(nond) — SC | 0.048 | 0.0026 | 281 | 18.60 | <.0001 |
|  | LS3(nond) — SL3 | 0.0068 | 0.0026 | 281 | 2.66 | 0.11 |
|  | LS3(nond) — SL4 | 0.028 | 0.0026 | 281 | 10.67 | <.0001 |
|  | LS4 — SC | 0.051 | 0.0018 | 281 | 28.12 | <.0001 |
|  | LS4 — SL3 | 0.0098 | 0.0018 | 281 | 5.55 | <.0001 |
|  | LS4 — SL4 | 0.031 | 0.0019 | 281 | 16.60 | <.0001 |
|  | SC — SL3 | -0.041 | 0.0018 | 281 | -23.21 | <.0001 |
|  | SC — SL4 | -0.020 | 0.0019 | 281 | -10.81 | <.0001 |
|  | SL3 — SL4 | 0.021 | 0.0018 | 281 | 11.56 | <.0001 |

**Table S4.** Treatment contrasts for analysis of weight, **Öland** **population** (log-transformed and averaged across sexes). For instars 3 and 4, planned contrasts were used to handle shared photoperiod regimes between treatment groups. For the pupal weights, all pairwise treatment contrasts were analyzed using Tukey’s method. Treatment abbreviations reflect the order of photoperiod regimes and in which instar the photoperiod changed: LC, long control; SC, short control; LS3, long to short in 3^rd^ instar; LS4, long to short in 4^th^ instar; SL3, short to long in 3^rd^ instar; SL4, short to long in 4^th^ instar.

| **Dev. stage** | **Contrast** | **Difference** | **SE** | **df** | **t** | **p** |
| --- | --- | --- | --- | --- | --- | --- |
| Third instar | Long days — short days | -0.0032 | 0.0088 | 612 | -0.36 | 0.72 |
| Fourth instar | Long days — short days | -0.016 | 0.011 | 591 | -1.49 | 0.14 |
|  | Long days — LS3 | 0.0024 | 0.013 | 616 | 0.19 | 0.85 |
|  | Short days — SL3 | 0.0053 | 0.013 | 560 | 0.43 | 0.67 |
| Pupa | LC-LS3 | 0.0010 | 0.014 | 582 | 0.091 | 0.99 |
|  | LC-LS4 | -0.0090 | 0.014 | 594 | -0.62 | 0.10 |
|  | LC-SC | -0.060 | 0.015 | 567 | -4.011 | 0.001 |
|  | LC-SL3 | -0.035 | 0.014 | 583 | -2.49 | 0.130 |
|  | LC-SL4 | -0.054 | 0.015 | 584 | -3.61 | 0.0044 |
|  | LS3-LS4 | -0.010 | 0.014 | 576 | -0.72 | 0.98 |
|  | LS3-SC | -0.061 | 0.015 | 549 | -4.11 | <0.001 |
|  | LS3-SL3 | -0.037 | 0.014 | 564 | -2.58 | 0.1035 |
|  | LS3-SL4 | -0.055 | 0.015 | 566 | -3.71 | 0.0031 |
|  | LS4-SC | -0.051 | 0.015 | 562 | -3.42 | 0.0086 |
|  | LS4-SL3 | -0.026 | 0.014 | 577 | -1.86 | 0.43 |
|  | LS4-SL4 | -0.045 | 0.015 | 580 | -3.031 | 0.031 |
|  | SC-SL3 | 0.024 | 0.015 | 549 | 1.66 | 0.56 |
|  | SC-SL4 | 0.0062 | 0.015 | 552 | 0.40 | 0.99 |
|  | SL3-SL4 | -0.018 | 0.015 | 566 | -1.24 | 0.82 |

**Table S5.** Treatment contrasts for analysis of weight, **Skåne** **population** (log-transformed and averaged across sexes). For instars 3 and 4, planned contrasts were used to handle shared photoperiod regimes between treatment groups. For the pupal weights, all pairwise treatment contrasts were analyzed using Tukey’s method. Treatment abbreviations reflect the order of photoperiod regimes and in which instar the photoperiod changed: LC, long control; SC, short control; LS3, long to short in 3^rd^ instar; LS4, long to short in 4^th^ instar; SL3, short to long in 3^rd^ instar; SL4, short to long in 4^th^ instar.

| **Dev. stage** | **Contrast** | **Difference** | **SE** | **df** | **t** | **p** |
| --- | --- | --- | --- | --- | --- | --- |
| Third instar | Long days — short days | 0.020 | 0.0091 | 537 | 2.16 | 0.031 |
| Fourth instar | Long days — short days | 0.021 | 0.011 | 541 | 1.89 | 0.060 |
|  | Long days — LS3 | 0.022 | 0.013 | 530 | 1.68 | 0.093 |
|  | Short days — SL3 | -0.018 | 0.014 | 531 | -1.35 | 0.18 |
| Pupa | LC-LS3 | 0.026 | 0.015 | 526 | 1.69 | 0.54 |
|  | LC-LS4 | 0.00034 | 0.016 | 527 | 0.022 | 0.99 |
|  | LC-SC | -0.0038 | 0.015 | 543 | -0.24 | 0.99 |
|  | LC-SL3 | -0.017 | 0.015 | 524 | -1.1 | 0.88 |
|  | LC-SL4 | -0.026 | 0.016 | 514 | -1.57 | 0.62 |
|  | LS3-LS4 | -0.026 | 0.015 | 521 | -1.67 | 0.55 |
|  | LS3-SC | -0.030 | 0.015 | 535 | -1.97 | 0.36 |
|  | LS3-SL3 | -0.043 | 0.015 | 516 | -2.84 | 0.05 |
|  | LS3-SL4 | -0.052 | 0.016 | 507 | -3.23 | 0.017 |
|  | LS4-SC | -0.0041 | 0.015 | 540 | -0.27 | 0.99 |
|  | LS4-SL3 | -0.017 | 0.015 | 520 | -1.13 | 0.87 |
|  | LS4-SL4 | -0.026 | 0.016 | 509 | -1.60 | 0.60 |
|  | SC-SL3 | -0.013 | 0.015 | 535 | -0.87 | 0.95 |
|  | SC-SL4 | -0.022 | 0.016 | 522 | -1.37 | 0.75 |
|  | SL3-SL4 | -0.0088 | 0.016 | 505 | -0.55 | 0.99 |

**Table S6.** Treatment contrasts for analysis of weight, **Stockholm** **population** (log-transformed and averaged across sexes). For instars 3 and 4, planned contrasts were used to handle shared photoperiod regimes between treatment groups. For the pupal weights, all pairwise treatment contrasts were analyzed using Tukey’s method. Treatment abbreviations reflect the order of photoperiod regimes and in which instar the photoperiod changed: LC, long control; SC, short control; LS3, long to short in 3^rd^ instar; LS4, long to short in 4^th^ instar; SL3, short to long in 3^rd^ instar; SL4, short to long in 4^th^ instar.

| **Dev. stage** | **Contrast** | **Difference** | **SE** | **df** | **t** | **p** |
| --- | --- | --- | --- | --- | --- | --- |
| Third instar | Long days — short days | 0.017 | 0.0094 | 631 | 1.89 | 0.059 |
| Fourth instar | Long days — short days | 0.014 | 0.011 | 572 | 1.22 | 0.25 |
|  | Long days — LS3 | 0.018 | 0.013 | 556 | 1.34 | 0.18 |
|  | Short days — SL3 | -0.0050 | 0.012 | 563 | -0.38 | 0.71 |
| Pupa | LC-LS3 | 0.036 | 0.016 | 546 | 2.26 | 0.21 |
|  | LC-LS4 | 0.029 | 0.017 | 540 | 1.75 | 0.50 |
|  | LC-SC | 0.016 | 0.016 | 563 | 1.02 | 0.91 |
|  | LC-SL3 | 0.003 | 0.016 | 549 | 0.20 | 0.99 |
|  | LC-SL4 | -0.032 | 0.016 | 561 | -1.98 | 0.35 |
|  | LS3-LS4 | -0.007 | 0.015 | 536 | -0.44 | 0.99 |
|  | LS3-SC | -0.020 | 0.014 | 562 | -1.40 | 0.73 |
|  | LS3-SL3 | -0.033 | 0.014 | 545 | -2.27 | 0.21 |
|  | LS3-SL4 | -0.068 | 0.015 | 559 | -4.55 | <0.001 |
|  | LS4-SC | -0.013 | 0.015 | 554 | -0.88 | 0.95 |
|  | LS4-SL3 | -0.026 | 0.015 | 539 | -1.70 | 0.53 |
|  | LS4-SL4 | -0.061 | 0.016 | 552 | -3.89 | 0.0016 |
|  | SC-SL3 | -0.013 | 0.014 | 568 | -0.90 | 0.95 |
|  | SC-SL4 | -0.048 | 0.015 | 581 | -3.29 | 0.014 |
|  | SL3-SL4 | -0.035 | 0.015 | 565 | -2.39 | 0.16 |
